# Oral Complications Associated with the Piercing of Oral and Perioral Tissues and the Corresponding Degree of Awareness among Public and Professionals: A Systematic Review

**DOI:** 10.3390/diagnostics13213371

**Published:** 2023-11-02

**Authors:** Seyed Ali Mosaddad, Sahar Talebi, Maryam Hemmat, Mohammadreza Karimi, Alireza Jahangirnia, Mostafa Alam, Kamyar Abbasi, Mohsn Yazadaniyan, Ahmed Hussain, Hamid Tebyaniyan, Reza Abdollahi Namanloo

**Affiliations:** 1 Student Research Committee, School of Dentistry, Shiraz University of Medical Sciences, Shiraz 71348-14336, Iran; mosaddad.sa@gmail.com (S.A.M.);; 2 Department of Conservative Dentistry and Bucofacial Prosthesis, Faculty of Odontology, Complutense University of Madrid, 28040 Madrid, Spain; 3 Research Committee, School of Dentistry, Isfahan University of Medical Sciences, Isfahan 14166-34793, Iran; 4 Independent Researcher, Tehran, Iran; 5 Department of Oral and Maxillofacial Surgery, School of Dentistry, Shahid Beheshti University of Medical Sciences, Tehran 19839-69411, Iran; 6 Department of Prosthodontics, School of Dentistry, Shahid Beheshti University of Medical Sciences, Tehran 19839-69411, Iran; 7 School of Dentistry, Edmonton Clinic Health Academy, University of Alberta, Edmonton, AB T6G 1C9, Canada; 8 Department of Science and Research, Islimic Azade University, Tehran 15847-15414, Iran; tebyan.hamid@yahoo.com; 9 Dentistry Department, Bogomolets National Medical University, 01601 Kyiv, Ukraine

**Keywords:** systematic review, piercing, health risk, oral health, prevention

## Abstract

This study systematically reviews the literature to evaluate the potential relationships between oral/perioral piercing and consequent oral complications in the corresponding society. The second objective was determining public/professional sectors’ awareness of the subject. This research followed PRISMA and Cochrane guidelines for conducting systematic reviews and searching scientific databases, including PubMed, Scopus, Cochrane, and Google Scholar, until April 2023. Cross-sectional, cohort, and case–control studies in English were deemed eligible. The methodological quality of the included studies was assessed using proper quality assessment guidelines. Of the 965 initial articles retrieved, 34 were considered suitable for qualitative synthesis after screening procedures and removing duplicates and irrelevant records. There appears to be an imbalance between the general public’s low and dentists’ high awareness. This draws attention to the shortage of professional and societal knowledge-sharing and education initiatives. Women were more than twice as likely as men to have oral piercings. Piercing usage had a low incidence among a cohort of students with a mean age of 16. Merely circumstantial evidence has indicated a plausible correlation between oral and perioral piercings and the emergence of secondary bacterial and fungal colonization, particularly periodontopathogenic bacteria and Candida albicans. Furthermore, several adverse consequences have been observed linked to various piercings—such as lip and tongue piercings. These include caries, gingivitis/periodontitis, dental fractures, enamel chipping/cracks, plaque buildup, bone loss, bleeding, inflammation, and swelling. Given the risks involved and the complications that might impair oral health, the prevalence of oral piercings is alarming. As a result, public health authorities need to firmly support initiatives to raise awareness of the risks associated with oral/perioral piercings. For piercers to enhance their expertise in this field, professional training is necessary because there is a shortage of knowledge on the possible adverse effects of piercings.

## 1. Introduction

**Figure 1:**
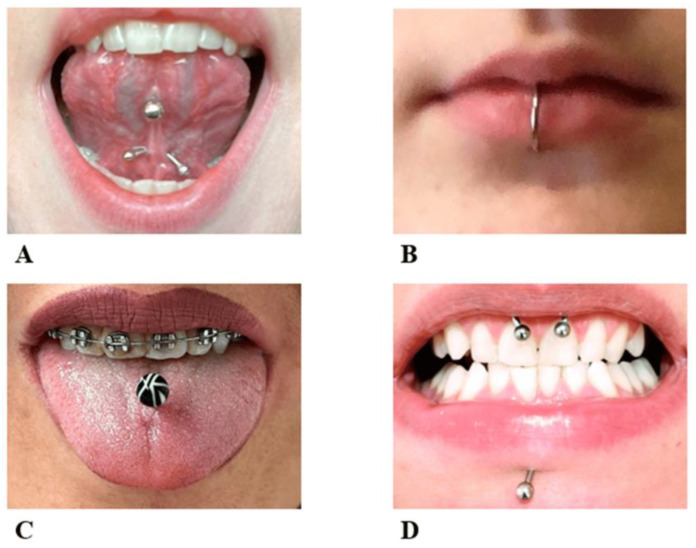
Different locations for oral piercing include (**A**) the lingual frenulum, (**B**) the lower lip, (**C**) the body of the tongue, (**D**) the upper labial frenulum, and labret piercing [15].

Piercing is a body ornamentation that different civilizations have accepted since the past as a manifestation of self-expression [1]. Today, body piercing is widely noticed among people, especially young people [2,3]. People with body piercings stated aesthetics, personal preference, and fit with the subculture as the main reasons for piercings [4]. Information sources available to individuals generally fail to provide information about health risk factors or other health-related issues [5,6]. The incidence of body piercing has been reported to range from approximately 17 to 70% in different individuals [7,8]. Oral piercings may be placed in various combinations on the lips, tongue, cheeks, or uvula (Figure 1). Oral piercing is unsafe, has positional and systemic hazards, and is associated with various complications [9]. Abnormal tooth wear, grinding, cracking, and gingival recession are late complications [10]. In addition, infection, abscess, and endocarditis can be considered severe systemic complications of oral piercing, which may even be life-threatening [11].

The tongue and lips were the two anatomical areas of the mouth where piercings were most frequently seen, and women were more likely than males to acquire oral piercings [12]. Gingival recession was mentioned as the most common complication. The central mandibular incisors reported the highest incidence of periodontitis and gingivitis. The frequency of tooth fracture was observed more in people with tongue piercing. Complications such as swelling and local inflammation after the piercing operation were among the injuries mentioned in the case reports that could threaten life. In addition, long-term piercing may cause gingival recession and tooth fracture [13]. There have been few systematic reviews or/and meta-analyses on the topic, each focusing on different aspects of oral/systemic health [14,15,16]. Their research primarily relied on case reports, which, due to their focus on individual patients, have limited statistical power. However, the dependability of the research was diminished by several investigations’ low quality and substantial heterogeneity across the studies [14,15,16]. In light of these apparent complications, clarifying how these injuries develop in oral piercing cases is necessary [17]. Therefore, this review aimed to systematically evaluate the literature for information on complications due to oral piercing.

## 2. Materials and Methods

The Preferred Reporting Items for Systematic Reviews and Meta-Analyses (PRISMA) and Cochrane Handbook for Systematic Reviews [18,19] were followed in this investigation. The protocol of this study was registered at the Open Science Framework (https://doi.org/10.17605/OSF.IO/MXKDC (accessed on 12 September 2023)). The central question of this research was: What are the oral health-related complications associated with oral piercings? The secondary research questions focused on the level of awareness regarding oral piercings-associated complications among public/health care professionals and their microbiological profile.

### 2.1. Search Strategy

The guidelines used for the search strategy are consistent with existing procedures for conducting systematic reviews. The four electronic databases, including PubMed, Scopus, Cochrane, and Google Scholar, were used to find papers that met the study’s criteria. In addition, electronic databases were searched from the inception until April 2023. The search terms used are presented in Supplementary Table S1.

### 2.2. Inclusion and Exclusion Criteria

The present study centered on the potential hazards posed by chemical, mechanical, and microbial factors in the context of piercing procedures and the awareness level regarding piercing-associated adverse effects among professionals/the public. The investigation analyzed primary research that presented evidence of these outcomes categorized into four main domains: public/professional awareness, periodontal/peri-implant complications, microbiological analyses, general and hard/soft tissue complications, and frequency of complications. The piercings in our definition encompassed those in the lips, cheeks, teeth, gingiva, buccal region, frenulum, uvula, tongue, and oral mucosa. The study incorporated case–control studies, cohorts, and cross-sectional studies, covering adolescent and adult populations and encompassing a wide range of piercing types. No limitations were imposed based on demographic factors such as age, height, weight, sexual orientation, race, or prior medical history. The studies included in the analysis were published from the beginning of the research field until April 2023. Articles written in English were included. In contrast, the analysis excluded all in vitro and in vivo research, case reports, interventional studies, hypotheses, correspondence, comments, letters to the editor, conference abstracts, editorials, and studies focused on exploring the association between oral piercings and general health risks.

### 2.3. Screening

**Figure 2:**
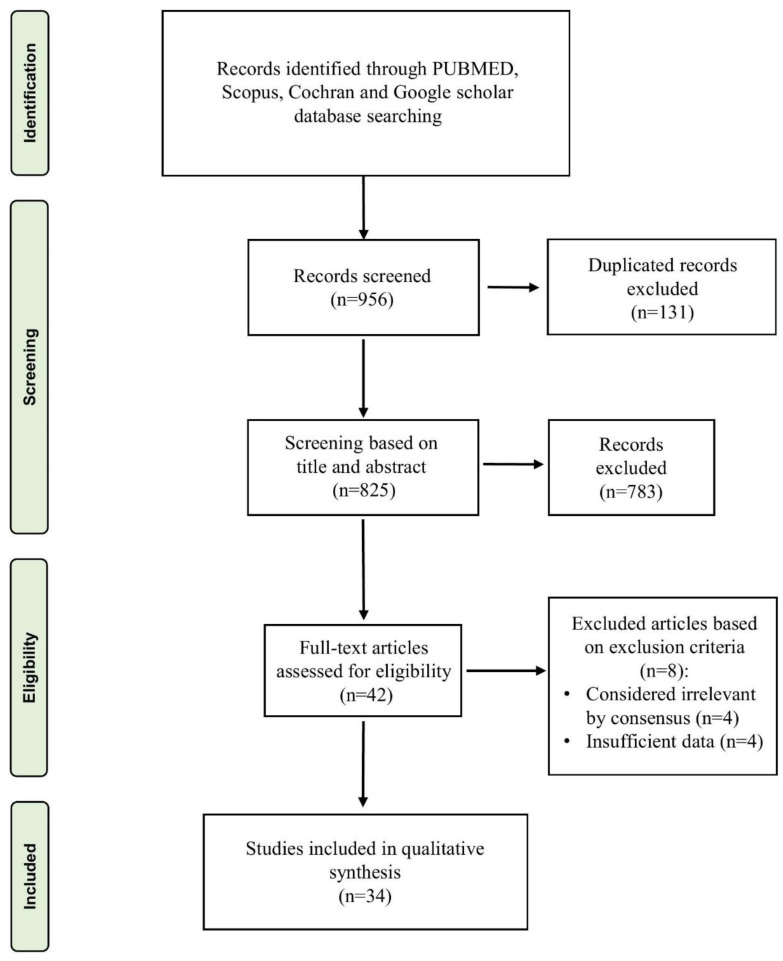
Flow charts for the studies were identified, displayed, and included in the study.

Following the articles’ retrieval from the database search, they were imported into the Endnote reference management software. The assessment of studies for potential eligibility was conducted by evaluating their title and abstracts for inclusion. Two authors (S.A.M and S.T.) independently assessed the title and abstract of each record to ascertain their suitability for inclusion in the review. Any disagreements between reviewers were resolved involving a third author (A.J.). Subsequently, the studies that satisfied the predetermined eligibility criteria underwent a thorough evaluation by scrutinizing the entire text document to ascertain their adherence to the established inclusion criteria. The review process is presented in Figure 2. The inter-reviewer reliability between the evaluators for the literature screening stage was determined using Cohen’s Kappa coefficient. Based on the frequency of precise agreements amongst reviewers, the kappa value (к) was calculated.

### 2.4. Data Extraction

Using established categories mutually agreed upon by all authors, specific information from each eligible record was extracted (Table 1). Bibliographic details, research methodology, patient demographics, piercing types, health assessments, research findings, and conclusions were collected. However, the extensive variability in outcomes related to the effects of piercings on oral health prevented us from conducting a meta-analysis.

### 2.5. Assessment of Quality

The risk of bias in the selected studies was assessed by two reviewers (S.T. and M.H.). The quality of case series studies was evaluated using a checklist including 20 criteria [20]. The answers “yes,” “no,” and “uncertain” were given to the cognitive method of each study. 0 to 2 “no” responses were considered low risk of bias, 3 to 5 “no” answers were considered moderate risk, 6 to 8 “no” responses were regarded as high risk, and more than 9 “no” responses were considered very high risk of bias. Additionally, the Newcastle-Ottawa Scale was devised to evaluate the quality of cohort, cross-sectional, and case–control studies [21].

## 3. Results

### 3.1. Study Design

The flow scheme of the literature search conducted for the systematic review is presented in Figure 2. The literature search yielded a total of 956 studies. Following the elimination of duplicates, the research mentioned above team proceeded to evaluate a total of 825 titles and abstracts of manuscripts. The process of full-text evaluation led to the exclusion of 783 out of 825 articles due to their failure to meet the established selection criteria. The systematic review included 34 [2,4,10,22,23,24,25,26,27,28,29,30,31,32,33,34,35,36,37,38,39,40,41,42,43,44,45,46,47,48,49,50,51,52] studies, subsequently evaluated for quality assessment. The Cohen’s Kappa coefficient (к = 0.91) showed almost perfect agreement between the reviewers in the selection process.

### 3.2. Study Characteristics

Of the 34 articles ultimately chosen, 4 were cross-sectional studies [31,37,41,52], 19 were case series [4,24,25,26,27,29,32,34,35,36,38,39,40,43,46,47,49,50,51], 9 were case–control studies [2,10,22,26,28,30,33,44,48], and 2 were cohort studies [42,45]. Research articles spanning from 2000 to 2022 were published, encompassing a diverse sample of both male and female participants (except [2]) within the age range of 16.14 ± 1.03 [46] and 38.2 ± 0.5 [22]. Three studies focused on microbiological analyses [28,29,30], four on bacterial plaque and infection alongside tissue complications [22,40,41,45], four only on awareness of side effects [23,24,27,36], twenty on pathological symptoms [2,10,25,26,31,32,35,37,38,39,41,42,43,44,45,46,47,48,49,50,51,52], one study on awareness and pathological symptoms [4] and one study also mentions the effects of depression caused by piercing [33]. Among the selected studies, eighteen papers cover the oral, peri- and intraoral cases [4,23,24,25,27,32,33,35,36,37,39,42,43,45,46,50,51], eleven just focused on tongue piercing [2,10,22,28,29,30,40,44,47,48,52], three on both lip and tongue piercing [26,34,49], two on lip piercing cases [31,41], and one study on both oral and facial piercing [38].

### 3.3. Quality Assessment

The Institute of Health Economics (IHE) checklist was utilized to evaluate the potential for bias in case series studies. According to the established criteria, the potential for bias was deemed low in 14 analyses [4,24,25,26,27,29,32,34,35,36,38,39,40,43], while four studies were found to have a moderate risk of bias [47,49,50,51]. One study was identified as having a high risk of bias [46]. The included case–control studies were classified according to the adjusted NOS, and four studies were considered fair quality [2,30,33,44], while the remaining five had good quality [10,22,26,28,48]. The two cohort studies under consideration were of fair quality [31,42,45]. Among the four cross-sectional studies included in the analysis, one was of poor quality [52], one was of fair quality [31], and the remaining two were of good quality [37,41] (Table 2, Table 3, Table 4 and Table 5).

### 3.4. Studied Outcomes

#### 3.4.1. Public/Professional Awareness

The purpose of the study by Covello et al., 2020 was to analyze people’s awareness of the side effects of oral piercing and to observe the side effects of piercing. Three hundred eighty-seven individuals with oral piercings were surveyed anonymously, while 70 participants underwent dental health and gingival recession examinations. The results of the analysis of the questionnaires showed that 46.8% of people did not know about the risks of piercing, 70.6% of people said that they were not aware of the gingiva problems that may arise, and 60.4% of the people said that they did not know about the risks of piercing to cause dental issues. Among the people under examination, 52.8% had poor oral health conditions, 42% had symptoms of generative gingivitis, 20% had 3 to 4 mm cavities, and 22% had tooth fracture(s) due to piercing [4]. The study of Junco et al. (2017) aimed to evaluate the effects of an educational program on dental students’ knowledge of oral piercing. They designed a training program for 66 dental students, during which dental students’ knowledge about oral piercing was evaluated before, immediately after, and 12 months after the training program by answering a questionnaire. The study’s findings showed a statistically significant difference regarding oral piercing knowledge between the groups of dental students before and after the educational intervention [23]. King et al. (2018) aimed to conduct a survey study to determine the knowledge and behavior of dentists toward patients with oral piercings. They collected this information from 200 dentists using a questionnaire. Only fifty-three dentists answered the questions. Of this number, 24.5% were very aware of the side effects of piercing. However, most dentists (73.6%) stated they obtained information empirically, and the recommendations provided varied significantly [24].

In a survey, Vozza et al. (2014) assessed people’s awareness of the local and systemic risks of mouth piercing. They asked 30 people with piercings to answer a 20-question questionnaire. 66.6% of people answered the questions. Only 20% of the people had enough information about the anatomy of the oral cavity, none of them knew about the anatomy of the tongue and gingiva, and only 10% said that a dental visit was necessary. Additionally, 40% of the respondents stated the need to take care of piercings [27]. Results from a case-series survey of people who have pierced their tongues or lips suggest that most piercing recipients did so to express their uniqueness and that more than half of piercing recipients have seen changes in their mouths and/or bodies [34]. Oberholzer et al. (2010) conducted a case series on individuals with intraoral piercing. The study found that a significant proportion of participants (59.4%) reported a lack of awareness regarding the potential complications associated with oral piercing. Within the past year, a notable proportion of respondents, precisely 24%, reported having undergone an intraoral piercing. Additionally, 17.2% of respondents indicated they had obtained such a piercing five to seven years before the survey [36].

#### 3.4.2. Periodontal/Peri-Implant Complications

The study by Ibraheem et al., 2022 aimed to evaluate the role of tongue piercing on periodontal and peri-implant health in adults. They divided people into two experimental (*n* = 48) and control (*n* = 49) groups, including people with and without tongue piercing. The required information was collected using a questionnaire. These people were examined regarding oral and dental health indicators such as entire mouth plaque and around the implant, gingival index, clinical attachment loss, and bone loss. Their results showed that in the experimental group, plaque index around the implant, gingival index, probing depth, and crestal bone loss were significantly higher in the anterior mandible [22]. Schmidt et al. (2019) aimed to evaluate the relationship between oral piercing and periodontal health or inflammation in periodontal patients. Their study included eighteen patients with tongue and lip piercings. This number had 14 tongue holes and seven lip holes. In patients with tongue piercings, the percentage of bleeding sites on probing, probing pocket depth ≥ 6 mm, clinical attachment loss ≥ 6 mm, and gingival recession ≥ 2 mm increased in teeth compared to teeth unaffected by piercing. In patients with lip piercing, the periodontal findings in the teeth close to the piercing were not significantly different from those unaffected [26]. In a cross-sectional case–control study, Ziebolz et al. (2020) investigated the oral health of patients with tongue piercing. They put 50 participants with tongue piercings and 50 without piercings into two experimental and control groups, respectively. The dental examination included missing- and filled-teeth-index and non-carious tooth defects. The periodontal examination had periodontal probing depth, bleeding on probing, and recession. The factors related to piercing and oral health-related quality of life were evaluated using questionnaires. People with tongue piercings suffered worse from missing- and filled-teeth-index, periodontal probing depth, bleeding on probing, and recession. In addition, a higher prevalence of tooth enamel cracks and dent-shaped scratches was observed in the piercing group. Most participants had tongue piercings, worse verbal behavior, insufficient cleaning of piercings, and, in 80% of cases, mass formation on the surface of the piercing, as well as oral health-related quality of life [10]. Gingival recession was more prevalent and severe in the case group than in the control group, according to research by Pires et al. (2010) conducted on the same group of piercing patients. Compared to those without tongue piercings, individuals with piercings were 11 times more likely to have a gingival recession in the anterior lingual mandibular region. Gingival recession in the anterior lingual mandibular area was associated with piercing use, increased age, male gender, and bleeding on probing [48].

#### 3.4.3. Microbiological Analyses

In a cross-sectional study, Ziebolz et al. (2019) investigated the prevalence of pathogenic periodontal bacteria in patients with tongue piercings compared to the control group. Fifty participants were placed in each group. They took samples from the piercing surface, periodontal pocket, and tongue and examined for the presence of 11 potential periodontal pathogenic bacteria. Most of the investigated bacteria were identified in the periodontal pocket of the piercing group compared to the control group, and a significant relationship was observed between the piercing surface and the periodontal pocket [28]. In a case-series study on 12 patients with tongue piercing, the study participants completed a questionnaire that provided specific information about their piercing characteristics, such as the type of material used, the duration of the piercing, their personal hygiene practices related to oral and piercing care, and their smoking status. The polymerase chain reaction (PCR) technique was employed to analyze the DNA of 11 periodontopathic bacteria. The microbiological samples were collected from the surface of the piercing jewelry located adjacent to the tongue hole. Results showed that Their tongue piercings had been in place for anything from 2 years to 8 years. The microbiological analysis showed an increased or considerably increased concentration of periodontopathic bacteria in all cases. The longer a piercing had been in place, the more noticeable the shift from bacteria with a moderate periodontopathic potential to microorganisms with a high periodontopathic potential occurred [29]. In 2010, Zadik et al. conducted a case–control study on the effects of tongue piercing. Young individuals who had just pierced their tongues had swabs obtained from the anterior lingual mucosa. In addition, a control group included people with facial piercings outside of the mouth. Light microscopy was used to examine *Candida* colonization. Chromogar samples that confirmed positive were re-cultured on *Candida* plates. People with tongue piercings were likelier to have *Candida* overgrowth than those with face piercings. Each colony had evidence of *Candida albicans*. The characteristics of current tongue ornament users and non-wearers were not substantially different. Multivariate research found that the only positive impacting factors on colonization were tongue piercing and smoking more than ten daily cigarettes [30]. Other case series on tongue piercings found that 28.8% of persons experienced lingual recessions and that 5% of people experienced at least one broken tooth. Only *Aggregatibacter actinomycetemcomitans* (Y4), Fusobacterium nudum species, and *Parvimonas micra* were found in samples obtained from piercing channels and studs, all associated with periodontitis. A sample made of stainless steel showed significantly higher levels of 67 of the 80 bacterial species examined compared to polytetrafluoroethylene and polypropylene holes [40].

#### 3.4.4. General and Hard/Soft Tissue Complications

**Figure 3:**
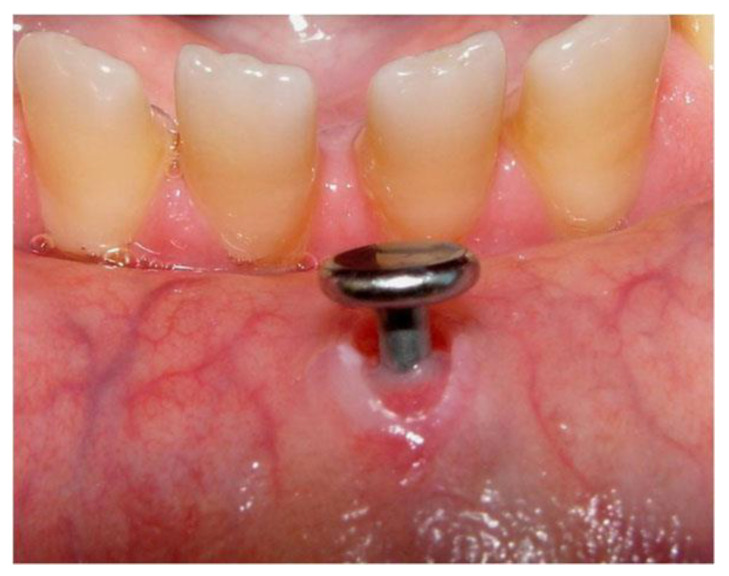
Mucosal damage around a labial piercing [53].

Ziebolz et al. (2012) conducted case–control research comparing people who had tongue piercings with a control group with comparable demographics to collect information regarding the timing and components of the piercing. During the dental exams, they looked for signs of dental caries, calculus, plaque, gingival diseases, enamel fissures, enamel fractures, and recessions. Researchers discovered that those with tongue piercings are more likely to acquire enamel fissures, enamel fractures, and lingual recessions. However, in the group that had their tongues pierced, enamel fissures, enamel fractures (Figure 3), and recessions were more prevalent than in the control group. The difference between the two groups was statistically significant [2]. Patients’ periodontal disease, dental health, and mucosal health were all assessed by Vilchez-Perez et al. in a cross-sectional study of lateral lower lip piercing. According to the data, the pierced side had less keratinized and linked gingiva and a higher prevalence of gingival recession. The canine and primary bicuspid teeth of most individuals were knocked out. More teeth broke or cracked on the piercing side than the unpierced side. Mucosal lining abnormalities were found in seven individuals [31].

**Figure 4:**
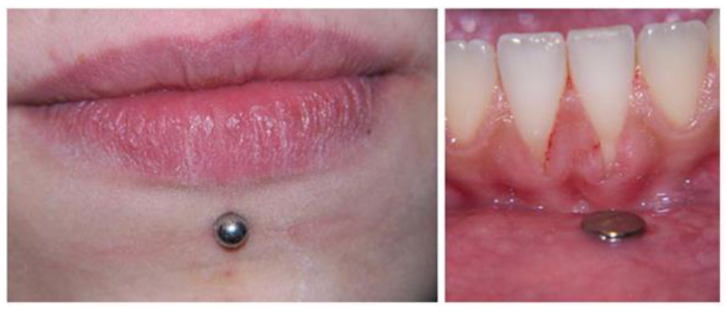
Piercing-associated gingival recession at the labial side of the mandibular anterior teeth [54].

**Figure 5:**
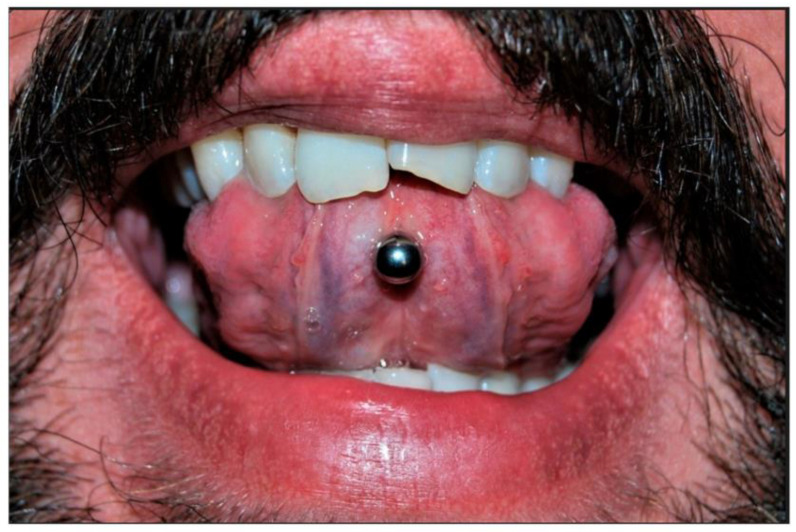
Enamel fracture of the maxillary left central incisor due to tongue piercing [4].

Pearose et al. conducted a case-series study on individuals with oral piercings using a questionnaire. The findings indicated that out of 508 respondents, only 49 individuals (10%) reported having an oral piercing. The piercing procedure was associated with various adverse effects, such as swelling, soreness, numbness, taste loss, bleeding, and pus formation. Oral piercings were given minimal attention or consideration. Oral injuries were frequently observed, particularly those affecting dentition [35]. A study on patients who had undergone oral and perioral piercing revealed that a minimum of one issue was observed in 96% of the sample immediately after the procedure or later. The most frequently observed adverse effects were mucosal atrophy, difficulties in eating or speaking, gingival recessions, tooth wear, enamel chipping or cracking, dentinal hypersensitivity, and excessive salivation [37]. Lateral lower lip piercings were also assessed by Kapferer et al., who found that four of the experimental teeth and one of the control teeth exhibited symptoms of mid-buccal regression. Canines and front teeth were the most often lost. Only one of the teeth in the test group suffered a chip, while all the teeth in the control group were unharmed. Plaque was much higher on teeth in the experimental group compared to the control group [41]. Case-control research comparing the dental health of 25 adults with and without labial and lingual piercings found that those with piercings were likelier to have uneven tooth wear and damaged teeth. Gingival recession (Figure 4) was also more severe in those with tongues or lips pierced than those without. Clinical attachment loss and pocket depth showed no significant differences between the two groups. There seems to be an association between piercing duration and dental abnormalities since 13 persons who had piercings for more than four years had a considerably greater frequency of tooth and periodontal disorders [44]. The most common dental problem identified by patients with tongue piercings was tooth chipping, according to cross-sectional research conducted by De Moor et al. (2000). Two teeth were chipped, and four cusps were shattered. There was a single case report of selective dental abrasion. Most patients had experienced an injury to the front of their tongues, called the lingual gingiva. Out of 15 participants, only two said they had any noticeable salivation. Patients did not notice any issues with their ability to chew or swallow food. There was a single recorded case of galvanic current production from an appliance [52]. Another study conducted by the same team in 2005 found that chipped teeth were the most often reported dental issue among patients with oral and perioral piercing. This problem was most common among those with tongues pierced (Figure 5). Gingival recession was seen in those who had lip piercings using studs. After surgery, patients had issues including swelling, bleeding, and infection [51].

#### 3.4.5. Frequency of Complications

Mejersjo et al. (2016) investigated the frequency of piercings and oral parafunction about the symptoms of temporomandibular disorders in adolescents. They included 124 high school students in their study, and the students answered questionnaire questions about different functions and symptoms of temporomandibular disorders. Of this number, 116 students underwent clinical examination of the temporomandibular system and tooth wear estimation. 14% of students had oral piercings. Female students had more headaches, severe symptoms, medical consultations, and used more painkillers. They also had more oral piercings than boys. Oral piercing was associated with symptoms of headache, muscle sensitivity, daily nail-biting, and tooth wear [25].

The number of teeth with caries, gaps, and restorations (DMF index) was assessed by Ventä et al. using the Beck Depression Inventory and other questionnaires. Just 3.4% of the population was entirely piercing-free. There were no significant variations in any DMF markers between the groups. Students who had their ears pierced were more likely to drool than their peers who did not. People in the study were more likely to be depressed, cigarette smokers, and drug users than the control group [33]. In a case study, Vieira et al. analyzed 42 cases comprising 39 young individuals who either had mouth piercings or had them removed because of difficulties. Most of the 29 individuals who had complications were affected by prolonged bleeding (69%), followed by pain (52.3%). Two cases of syncope were documented. Patients had post-insertion complications such as pain and swelling in 97.6% of cases. Problems with the surrounding tissues, such as tooth soreness and tongue lacerations, were observed in 33.3% and 31% of cases, respectively [32]. By conducting two case-series on oral and facial piercings in 2006, López-Jornet et al. discovered that pain was the most often reported condition, occurring in 60% of patients, followed by inflammation (34.3%), bleeding (24%), tooth fractures or fissures (20%), and gingival damage (26.7%). Another research found that recessions of the gingiva were present in 23 individuals (23.5%), most often around the mandibular incisors. Thirteen (13.3%) cases suffered tooth structural damage [38,39]. According to retrospective cohort research conducted by Inchingolo et al. on 108 cases of oral piercing, there were no significant complications. Even though all patients said they had followed the piercers’ instructions, 90% had postoperative bleeding within 12 h, 80% experienced perilesional edema for 3.5 days after the piercing, and 70% experienced chronic mucosal atrophy [42]. Hickey et al. (2010) conducted a case series on people who have oral piercings. They found that only around a quarter of them heal without incident, with the risk of complications varying wildly depending on how near to the mouth the piercing was. The gingival recession affected 8.5% of those aware of issues, while tooth-chipping affected 6.9%. Among this sample, 52.9% blamed titanium, 23.5% blamed stainless steel, and 9% blamed Teflon for their economic downturn, whereas 35.7%, 42.5%, and 14.3% blamed titanium, stainless steel, or Teflon for their tooth chipping [43]. Lip and tongue wounds accounted for 46% of all reported instances in a cohort analysis of 24,349 patients with Oral piercings, whereas tooth injuries accounted for 10%. Puncture wounds to soft tissue ranked second (29%), behind infections (42%). Most people who go to the emergency room do so because the mucosa surrounding their oral piercings has become too large (39%). Inpatient care was seldom required [45].

Furthermore, just 3.6% of the children tested had oral piercings, with 69.70% attending public schools and 30.30% attending private ones, according to a case-series by dental patients with oral piercings. Men outweigh women somewhat (54.55% to 45.45%). Tongues were pierced by a large majority (66.6%). In 74.3% of cases, piercing caused the complications and changes predicted for its use [46]. Campbell et al. found no lingual recession or tooth-chipping cases among 52 individuals whose tongues were pierced between 0 and 2 years before. Linguistic regression of the mandibular central incisors was seen in 50% of the subjects who wore long barbells for 2+ years. Patients with their tongues pierced for four years or longer had a 47% incidence of chipped molars or premolars [47]. In a case study by Kieser et al. (2005), patients who received tongue or lip piercings were given a questionnaire after an intraoral examination. Participants had their tongues pierced at a rate of 76.7%, their lips pierced at a rate of 34.9%, or both at a rate of 11.6%. Only four of the piercings were performed by a qualified medical practitioner—thirty-four percent of those who had piercings reported complications. Eighty percent of persons with labial piercings showed signs of gingival recession at one or more piercing sites, while almost one-third of those with tongue piercings showed signs of recession. There was a statistically significant increase in the risk of lingual recession beyond the age of 14 of 1.17 times. Labial recession became more common as people became older, and the number of affected locations increased with age, although lingual recession was not predicted by age. They found no evidence linking piercings to increased tooth wear or damage [49]. Before a dental exam, Levin et al. surveyed people who had oral piercings to learn more about their experiences with the procedure, their knowledge of potential risks, and the frequency with which they had complications. Most people with oral piercings acquire their linguae pierced; 79 people (or 20.3%). After getting their ears pierced, 41 (51.9%) and 36 (45.7%) people suffered swelling and bleeding. Of the whole group, 57.8 percent, or 225 persons, were unaware that intra-oral piercings might be dangerous. 11 (or 13.9%) of the piercings were found to have fractured teeth upon closer scrutiny. Gingival recessions were identified in twenty-one individuals (or 26.6%), most often around the mandibular incisors [50].

## 4. Discussion

This study was a systematic review of oral piercings. The widespread acceptance of oral piercing leads to increased complications, and relevant professionals must be prepared to face such situations [11,32,55]. The present systematic review aimed to obtain information on complications related to oral piercings to assess the degree of risks associated with piercings accurately and to gather data on the degree of public and professional awareness regarding oral-piercing-related side effects. Dental injuries mean defects such as broken, cracked, and worn teeth. A previous systematic review shows that the risk ratio of gingivitis in people with lip and tongue piercings was 4.14 and 2.77 times higher than in people without piercings, respectively [56]. In addition, the risk ratio of tooth damage in people with tongue and lip piercings was 2.44 and 4.14 times higher than in those without piercings [56]. During the past years, a targeted orientation towards evidence-based medicine has been created via randomized controlled trial studies, with the highest confirmatory value for assessing the effectiveness of the type of intervention [57,58,59,60]. However, the focus of this review was on the evaluation of non-randomized trial studies. The studies identified in the literature search were exclusively non-randomized observational studies. However, studies that aim to calculate a risk factor cannot be randomized because exposing people to potentially harmful risk factors is considered unethical [61]. Therefore, observational studies quantitatively estimate the complications of oral piercing as a practical intervention without using randomization methods to assign subjects to comparison groups.

This review describes case–control studies as comparing groups of the same population with and without a specific desired outcome to investigate the relationship between exposure to an intervention and the outcome [62]. Based on these definitions, four case–control and five case-series studies were included. The results of the study by Covello et al. determined that there is not enough awareness about the complications caused by piercing and the correct methods of maintaining oral piercings, and oral piercings can be considered a potential risk to oral and dental health. Therefore, periodic examinations by specialists and dentists can effectively prevent and reduce the complications caused by oral piercing [4]. The study of Ibraheem et al. concluded that tongue piercing can increase the probability of periodontal diseases around the implant, especially in the anterior mandibular part [22]. Junco et al. concluded that the oral piercing educational intervention favored dental students, especially among those more engaged in the learning process [23]. The review results by King et al. show that dentists do not know enough about the risks and preventive recommendations for patients with piercings [24]. Mejersjo et al. stated in their study that there is a relationship between gingival health, nail-biting, and mouth piercing and symptoms of temporomandibular disorders [25]. Schmidt et al. stated that tongue piercing could negatively affect the periodontal conditions of the teeth close to the piercing [26]. Vozza et al. noted that the participants’ awareness of the potential risks of oral piercing is deficient [27]. Ziebolz et al. stated that the tongue-piercing surface should be considered an important ecological site and reservoir for periodontal pathogens [28]. Ziebolz et al. concluded that patients with tongue piercing lack good dental and periodontal health, so it is necessary to pay more attention to patients who use tongue piercing in dentistry [10]. Additionally, the same authors showed that prolonged usage of tongue jewelry might result in periodontopathogenic bacteria colonization at the piercing site if appropriate oral hygiene is not practiced [28]. The oral health concerns associated with piercings and the need for regular cleanings with appropriate disinfectant should be known to prospective and current pierces. The research also revealed that tongue piercings are not without their drawbacks. Tongue piercings have been linked to an increase in the prevalence of dental problems, such as cracks and fractures in the enamel and recessions of the gingiva around the teeth, especially in the lingual region of the mandibular incisors. So, since most people who have their tongues pierced are young adults, they should be the primary target of anti-piercing campaigns [47]. The second study proved how many people already know: most piercings are unhealthy for the individual having them. So, dentists should warn patients who receive mouth piercings to be ready for any potential problems [34]. Additionally, in two case-series studies on oral and face piercings, López-Jornet et al. emphasized the need to caution patients about the risks associated with intra-oral and facial piercings [38,39]. Although the number of teenagers getting oral piercings is small, Pearose et al. found that those who did so typically did so without their parents’ knowledge or consent and had signs of infection. Correspondingly, In the sample of high school students studied, 3.6% had oral piercings done, and those who did were more likely to have minor health problems [46].

Giuca et al. indicated that gingival recession is a common side effect of tongue and lip piercings. The more extended tongue and lip piercings are kept in place, the greater the likelihood and severity of dental anomalies, gingival recession, attachment loss, and probing depth of adjacent teeth. Rates of gingival recession may be traced back to the shape of dental decorations [44]. Additionally, results indicated that gingival recession and the amount of keratinized, attached gingiva are exacerbated by lateral lower lip piercings. So, using such devices has also been connected to tooth damage. Another report indicated that people with lower lips pierced laterally are more likely to develop plaque on their teeth close to the piercing [41]. In contrast, another study showed that an ornament in a tongue piercing was not associated with a higher incidence of Candida albicans colonization. Parallel to one another, The minimal number of germs in the piercing channels indicates that having the tongue pierced poses little risk of developing an oral infection. Studs made of polytetrafluoroethylene or polypropylene were shown to be less likely to be colonized by bacteria than those made of steel. Staphylococci on the steel or titanium stud may suggest an increased risk for problems if the piercing channel is polluted. However, Vieira et al. recommended that those with oral piercings only do so under the supervision of qualified doctors and continue to acquire frequent dental exams to catch any issues early [43]. Psychologists need to pay attention to research that found those with oral piercings had a much greater risk of smoking, illicit drug use, and depression than those without. Moreover, dentists should correct the widespread ignorance about the dangers of mouth piercing [36].

Interestingly, having a medical professional do the piercing reduces the potential for infection [42]. According to research by Gill et al., dentists in emergency rooms may need to address damage to both hard and soft tissues caused by oral piercings [45]. Our findings suggest that dentists should be more active in warning patients about the potential health consequences of getting a tongue or mouth piercing. In light of these considerations, individuals with oral piercings must maintain good oral hygiene practices, including brushing and flossing regularly and avoiding foods and drinks that irritate the piercing. They should also be aware of the adverse signs and seek prompt medical attention if they experience any symptoms. Ultimately, the decision to acquire an oral piercing should be carefully considered and discussed with a healthcare professional to ensure that it is performed safely and with minimal risk to overall health and well-being.

### Biases and Potential Confounders

Non-randomized studies are more likely to be biased than randomized studies. Therefore, the results should be interpreted cautiously, and attention should be paid to the possibility of selection bias [63]. Estimates of potential confounders indicate the degree of heterogeneity between studies. For example, the oral piercing may be accompanied by gingival recession. Still, this observed complication may have another origin, such as plaque inflammation, toothbrush trauma, smoking [64], caries, high lingual frenum [65], periodontal biotype [9,64], keratinized gingival height [31], and oral hygiene [2]. However, possible confounding factors, such as age, sex, type and length of piercing, location, and position of piercing, previous trauma, and the individual’s behavior in the piercing movement, can be relevant to the interpretation of this review. 

## 5. Conclusions

Considering the limitations of this study, these results can be mentioned. The severity of piercing complications is related to the location of the piercing inside the oral cavity. Piercing can be one of the causes of tooth cracks and buccal depression in teeth that are in direct contact with the piercing. Analysis was significantly high in both types of lip and tongue piercings. From the point of view of oral health specialists, the popularity of oral piercing can be worrying due to the number of oral complications and risks.

## Figures and Tables

**Table 1:** Baseline Characteristics of the included studies.

Study	Design (Male/Female Ratio)	Population	No. of Cases (Age Mean ± SD)	Piercing	Evaluation	Outcome	Conclusion
Covello et al. (2020)	Case-series (NA)	Adolescents with oral piercing	387 (NA)	Oral	They asked 387 people with oral piercings to complete a questionnaire anonymously. Additionally, 70 people were examined regarding dental health and gingiva recession.	The results of the analysis of the questionnaires showed that 46.8% of people did not know about the dangers of piercing, 70.6% of people said that they were not aware of the gingiva problems that may arise, and 60.4% of the people said that they did not know about the dangers of piercing to cause dental issues. Among the people under examination, 52.8% had poor oral health conditions, 42% had symptoms of generative gingivitis, 20% had 3 to 4 mm cavities, and 22% had tooth fracture(s) due to piercing.	There is not enough awareness about the complications caused by piercing, and the correct methods of maintaining oral piercings and oral piercings can be considered a potential risk to oral and dental health. Therefore, periodic examinations by specialists and dentists can effectively prevent and reduce the complications caused by oral piercing.
Ibraheem et al. (2022)	Case (15 (31.25%)/33 (68.75%)) control 19 (38.77%)/30 (61.23%)	Adolescents with oral piercing	48 (38.2 ± 0.5)/49 (37.5 ± 0.2)	Tongue	They divided people into two experimental (48) and control (49) groups, including people with and without tongue piercing. The required information was collected via a questionnaire.	These people were examined regarding oral and dental health indicators such as entire mouth plaque and around the implant, gingival index, clinical attachment loss, and bone loss. Their results showed that in the experimental group, plaque index around the implant, gingival index, probing depth, and crestal bone loss were significantly higher in the anterior mandible.	Tongue piercing can increase the probability of periodontal diseases around the implant, especially in the anterior mandibular part.
Junco et al. (2017)	Case (25 (37.9%)/41 (62.1%)) Control (11 (45.8)/13 (54.2))	dental students	66 (21.6 ± 1.7)/28 (23.0 ± 1.4)	Oral	They designed a training program for 66 dental students, during which dental students’ knowledge about oral piercing was evaluated before, immediately after, and 12 months after the training program by answering a questionnaire.	The study’s findings showed a statistically significant difference between the groups of dental students before and after the educational intervention.	The oral piercing educational intervention had a favorable effect on dental students, especially among those more engaged in the learning process.
King et al. (2018)	Case-series (NA)	dental students	53 (NA)		They collected this information from 200 dentists via a questionnaire. Only fifty-three dentists answered the questions.	Of this number, 24.5% were very aware of the side effects of piercing. However, most dentists (73.6%) stated that they obtained information empirically, and the recommendations provided varied significantly.	Dentists do not know enough about the risks and preventive recommendations for patients with piercings.
Mejersjo et al. (2016)	Case-series (71 (57.25%)/53 (47.75%))	High school students	124 (18.1 ± 2.9)	Oral	Students in their study answered questionnaire questions about different functions and symptoms of temporomandibular disorders. Of this number, 116 students underwent clinical examination of the temporomandibular system and tooth wear estimation.	They included 124 high schools. 14% of students had oral piercings. Female students had more headaches, more severe symptoms, more medical consultations, and used more painkillers. They also had more oral piercings than boys. Oral piercing was associated with symptoms of headache, muscle sensitivity, daily nail-biting, and tooth wear.	There is a relationship between gingival health, nail-biting, and mouth piercing and symptoms of temporomandibular disorders.
Schmidt et al. (2019)	Case-series (4 (22.2%)/14 (77.8%))	Adolescents with oral piercing	18 (28.3 ± 7.7)	Tongue, lip, tongue, and lip	Their study included eighteen patients with tongue and lip piercings. This number had 14 tongue holes and seven lip holes.	In patients with tongue piercings, the percentage of bleeding sites on probing, probing pocket depth ≥6 mm, clinical attachment loss ≥6 mm, and gingival recession ≥2 mm increased in teeth compared to teeth unaffected by piercing. In patients with lip piercing, the periodontal findings in the teeth close to the piercing were not significantly different from those unaffected.	Tongue piercing could negatively affect the periodontal conditions of the teeth close to the piercing.
Vozza et al. (2014)	Case-series (NA)	Adolescents with oral piercing	30 (NA)	Oral	They asked 30 people with piercings to answer a 20-question questionnaire.	66.6% of people answered the questions. Only 20% of the people had enough information about the anatomy of the oral cavity, none of them knew about the anatomy of the tongue and gingiva, and only 10% said that a dental visit was necessary. Additionally, 40% of the respondents stated the need to take care of piercings.	The participant’s awareness of the potential risks of oral piercing is deficient.
Ziebolz et al. (2019)	Cross-sectional (Case-control) (20%/80%)	Adolescents with oral piercing	50 NA/NA (28.3 ± 7.1/28.2 ± 7.1)	Tongue	Fifty participants were placed in each group. They took samples from the piercing surface, periodontal pocket, and tongue and examined for the presence of 11 potential periodontal pathogenic bacteria.	Most of the investigated bacteria were identified in the periodontal pocket of the piercing group compared to the control group, and a significant relationship was observed between the piercing surface and the periodontal pocket.	The tongue-piercing surface should be considered an important ecological site and reservoir for periodontal pathogens.
Ziebolz et al. (2020)	Case-control	Adolescents with oral piercing	50/50	Tongue	They put 50 participants with tongue piercings and 50 without piercings into two experimental and control groups, respectively. The dental examination included missing- and filled-teeth-index and non-carious tooth defects. The periodontal examination had periodontal probing depth, bleeding on probing, and recession. The factors related to piercing and oral health-related quality of life were evaluated using questionnaires.	People with tongue piercings suffered worse from missing- and filled-teeth-index, periodontal probing depth, bleeding on probing, and recession. In addition, a higher prevalence of tooth enamel cracks and dent-shaped scratches was observed in the piercing group, and most of the participants had tongue piercings, worse verbal behavior, insufficient cleaning of piercings, and in 80% of cases, mass formation on the surface of the piercing, as well as oral health-related quality of life.	Patients with tongue piercing lack good dental and periodontal health, so paying more attention to patients who use tongue piercing in dentistry is necessary.
Ziebolz et al. (2009)	Case-series (7 (58.33%)/5 (41.64%))	Adolescents with tongue piercings	12 (24.0 ± 2.8)	Tongue	Participants filled out a particular questionnaire detailing their piercing details, including the material used, the length of time the device was in place, their personal oral and piercing cleanliness practices, and whether or not they smoked. The DNA of the 11 periodontopathogenic bacteria was also analyzed by polymerase chain reaction (PCR) from microbiological samples obtained from the surface of the piercing jewelry next to the tongue hole.	The duration of their tongue piercings ranged from 2 years to 8 years. According to the microbiological examination, all instances had an elevated or significantly elevated concentration of periodontopathogenic bacteria. It became clear that the transition from bacteria with a moderate periodontopathogenic potential to microorganisms with a high periodontopathogenic potential was more prominent the longer a piercing had been in situ.	In the absence of good oral hygiene, prolonged use of tongue jewelry might lead to the colonization of periodontopathogenic bacteria at the piercing site. It is essential to educate both prospective and present piercers about the potential for adverse consequences, including risks to oral health, and on the requirement of frequently cleaning piercing jewelry with a CHX solution or another suitable disinfectant.
Ziebolz et al. (2012)	Case (46 (100%)/0 (0%))-control (46 (100%)/0 (0%))	Adolescents with tongue piercings	46 (22.2)/46 (22.1)	Tongue	Those with TP from the German Federal Armed Forces (group TP) and a control group with similar demographics (group C) volunteered for the research. It was recorded how long TP was left in place, where it was found, and what it was made of. During their dental checkups, they checked for caries, calculus, plaque, gingival disease, enamel fissures (EF), enamel fractures (EC), and recessions (R).	Enamel fissures, enamel fractures, and lingual recessions are more common in those who have tongue piercings. However, compared to the control group, the EF, EC, and R number was more significant in the tongue-piercing group. There was a statistically significant (*p* < 0.001) gap between the two groups.	This case–control research, within its scope, has shown that tongue piercing has negative long-term consequences. Enamel fissures, enamel fractures, and gingival recessions (particularly in the lingual area of mandibular incisors) were shown to be much more common in those who had tongue piercings.
Zadik et al. (2010)	Case (NA)-control (NA)	Adolescents with tongue piercings	115 (20.4 ± 1.6)/86 (20.7 ± 1.7)	Tongue	Swabs were taken from the anterior lingual mucosa of young people who had just pierced their tongues. Additionally, patients with non-intra-oral face piercings served as a control group. Colonization by *Candida* was studied by light microscopy. Chromagar *Candida* plates were used to re-cultivate the specimens that tested positive.	Tongue-pierced people were more likely to be colonized with *Candida* than facially pierced people. *Candida albicans* were found in every single colony. Current tongue ornament wearers and non-wearers did not vary significantly from one another. Multivariate analysis revealed that tongue piercing and smoking more than ten cigarettes daily were the only significant positive influencing variables on colonization.	There was no correlation between the presence or absence of an ornament in a tongue piercing and an increased risk of *Candida albicans* colonization.
Vilchez-Perez et al. (2009)	Cross-sectional (11 (22%)/39 (78%))	Adolescents with lip piercings	50 (21.3 ± 4.4)	Lateral lower lip	Patients had their piercing and non-piercing sides examined for periodontal disease, dental health, and mucosal health.	Women made up the vast majority (78%) of piercing enthusiasts. Gingival recession was more common, and the quantity of keratinized and connected gingiva was less on the piercing side. Most people lost their canine and initial bicuspid teeth. The piercing side had double the rate of tooth fractures and cracks compared to the non-pierced side. Seven individuals had abnormalities in their mucosal lining.	Piercing the lower lip on the side increases gingival recession and decreases the quantity of keratinized, connected gingiva. These accessories are also linked to broken or cracked teeth.
Vieira et al. (2011)	Case-series (13 (33.3%)/26 (66.7%))	Adolescent with oral piercings	39 (NA)	Oral	Complications from oral piercings were examined in 42 instances, including 39 young people who were now or formerly pierced in the mouth.	Twenty-nine patients had immediate problems, the most common of which were prolonged bleeding (69%) and discomfort (52.3%). There were two reported occurrences of syncope. In 97.6% of instances, patients reported late problems such as discomfort or edema at the insertion site of the piercing. In 33.3% and 31% of cases, respectively, problems involving the surrounding tissues, such as dental discomfort and tongue lacerations, were reported.	Anyone considering getting an oral piercing should be informed of the risks involved, most of which are limited to the immediate area. Those interested in being pierced should only do so under trained specialists’ care and maintain regular dental checkups to ensure early diagnosis of any potential complications.
Ventä et al. (2005)	Case-control (49 (21%)/185 (79%)	First-year university students with oral piercing	234 (20.6 ± 0.6)	Oral	Using the Beck Depression Inventory and other questionnaires, they measured the number of teeth with decay, gaps, and restorations (DMF index).	3.4% of the population has an oral piercing. There were no significant differences between the groups in any of the DMF indicators—students who had piercings tended to drool more often than their peers who did not. The study group had much higher rates of cigarette and illegal drug use, as well as depression, than the control group.	Oral piercings need special attention to aftercare because of the potential for oral complications.
Saquet et al. (2009)	Case-series (NA)	Adolescents with oral piercings	51 (NA)	Tongue or lip	Fifty-one people who have had oral piercings were surveyed.	Most respondents who got piercings in their mouths said they did so to show their individuality. Over half of those who did so reported experiencing oral and/or general changes due to piercings.	Most piercings are bad for the person getting them; thus, dentists should advise their patients who have oral piercings to be prepared for any complications and provide them the attention they need to avoid serious health issues.
Pearose et al. (2006)	Case-series (NA)	High school adolescents obtaining an oral piercing	508 (NA)	Oral	Five high schools were polled by questionnaire.	Only 49 out of 508 respondents (10%) reported having an oral piercing. Swelling, soreness, numbness, taste loss, bleeding, and pus were some of the side effects of the piercing. Little to no care was given to oral piercings. Injuries to the mouth, especially the teeth, were prevalent.	Researchers found that although the number of adolescents undergoing oral piercings was low, those who did so without parental permission often had infection symptoms.
Oberholzer et al. (2010)	Case-series (55 (22%)/195 (78%))	South African adults with intraoral piercings	250 (19.6 ± 5.3)	Intraoral	They were given a questionnaire to fill up with their knowledge of oral piercing risks. Then, two qualified dentists checked them out to see if there were any problems with their teeth from the piercing.	Most responders (59.4%) said they had no idea oral piercing may cause difficulties. In the recent year, 24% of respondents had an intraoral piercing, while another 17.2% got one between the ages of 5 and 7 years ago.	The dentistry community should address the widespread lack of knowledge about the risks of mouth piercing.
Lorenzini et al. (2008)	Cross-sectional (NA)	People having oral and perioral piercing	44 (NA)	Oral and perioral	Any person was given a complete physical examination and had their medical and dental histories recorded.	Oral and perioral piercings were associated with at least one problem in 96% of the sample, either immediately after the procedure or later on. Mucosal atrophy, difficulties eating or speaking, gingival recessions, tooth wear, enamel chipping or cracking, dentinal hypersensitivity, and excessive salivation were the most common adverse effects.	The observed problems are consistent with those described in the published literature. The authors noticed a correlation between the number of times people had mouth piercings and how long those piercings lasted.
López-Jornet et al. (2006)	Case-series (27 (18.7%)/43 (81.3))	Healthy individuals with oral and facial piercings	70 (17.08 ± 2.61)	Oral and facial	A standardized methodology was implemented to evaluate potential side effects (such as inflammation, discomfort, or tooth changes).	Pain was the most common reported problem, occurring in 60% of patients, followed by inflammation (34.3%), bleeding (24%), tooth fractures or fissures (20%), and gingival damage (26.7%).	Pain, swelling, and dental issues are all things you might expect after getting your tongue pierced.
López-Jornet et al. (2006)	Case-series (29 (29.9%)/68 (70.1%))	Individuals with intra-oral piercings	98 (20.06 ± 4.75)	Intraoral	The potential side effects were evaluated by dental examination.	In 23 patients (23.5%), recessions of the gingiva were seen, most often around the mandibular incisors. Thirteen instances (13.3%) had tooth structural damage.	Therefore, it is important to warn patients about the potential dangers of intra-oral piercings to their teeth and gingiva.
Kapferer et al. (2011)	Case-series (12 (15%)/68 (85%))	Individuals with intra-Tongue piercings	85 (22.74 ± 4.47)	Tongue	Bacterial samples were taken from tongue piercings constructed of various materials and analyzed using checkerboard deoxyribonucleic acid-deoxyribonucleic acid hybridization to determine the microbial differences between the materials.	There were 61 lingual recessions reported by 28.8% of participants, and 5% had at least one chipped tooth. Samples taken from studs and piercing channels seldom included periodontitis-related bacteria, except for *Aggregatibacter actinomycetemcomitans* (Y4), *Fusobacterium nucleatum* species, and *Parvimonas micra*. Compared to polytetrafluoroethylene and polypropylene perforations, a sample from stainless steel revealed considerably more significant levels of 67 of the 80 bacterial species studied.	There is little chance of contracting an oral infection after getting your tongue pierced, as shown by the low bacterial numbers in the piercing channels. Steel studs were more conducive to biofilm formation than polytetrafluoroethylene or polypropylene studs, indicating that the latter may be more resistant to bacterial colonization. If the piercing channel is contaminated, the presence of *Staphylococci* on the steel or titanium stud may indicate a higher risk for complications.
Kapferer et al. (2012)	Cross-sectional (NA)	Nondental setting population with lateral lower lip piercing	47 (NA)	Lateral lower lip	Full-mouth plaque and bleeding indexes, probing depth, recession, clinical attachment level, periodontal biotype, assessment of hard tissues, occlusal damage, stud features, and mucosal inspection and palpation were all part of the comprehensive clinical examination.	Four of the experimental teeth and one of the control teeth showed signs of mid-buccal recession. Most people lost their canines and front teeth. One of the test teeth chipped, while none of the control teeth did. Teeth in the experimental group had substantially more plaque than teeth in the control group.	Plaque buildup on teeth next to piercings is much more significant in those who have had their lower lips pierced laterally. Some people have tooth chipping or buccal recession after a lateral lower lip piercing.
Inchingolo et al. (2011)	Retrospective (74 (69%)/34 (31%))	Individuals who had an oral piercing performed by a healthcare professional	108 (NA)	Oral	Clinical examinations were performed on all of the patients to detect the occurrence of any late problems.	No severe problems occurred among the 108 individuals. All patients claimed that they had followed the piercers’ recommendations, yet 96% of them still had postoperative local issues, such as bleeding within 12 h of the piercing (90%), perilesional edema for 3.5 days after the piercing procedure (80%), and persistent mucosal atrophy (70%).	There are fewer risks and fewer infection opportunities with a medical practitioner doing the piercing.
Hickey et al. (2010)	Case-series (55 (27.36%)/146 (72.64%))	Individuals with oral piercing	201 (22.7)	Oral	They were asked to complete a questionnaire on what they knew about the dangers of oral piercings.	Only about a quarter of piercings go well, with the complication rate varying widely depending on how close the piercing was to the mouth. 8.5% of the group with knowledge of difficulties had a gingival recession, while 6.9% experienced chipped teeth. The recession was linked to titanium, stainless steel, and Teflon in 52.9%, 23.5%, and 9% of this population, whereas chipped teeth occurred in 35.7%, 42.5%, and 14.3%.	Complications happened a lot. More research, including dental checkups and public education, is required.
Giuca et al. (2012)	Case (11 (44%)/14 (56%))-control (11 (44%)/14 (56%))	Patients with a minimum of one labial or tongue piercing	25 (23.4 ± 3.6)/25 (NA)	Labial and tongue	Each patient was asked to complete a questionnaire and undergo a physical examination.	Labial and lingual piercing individuals were likelier to have irregular tooth wear and chipped teeth. In addition, those who’d had their tongues or lips pierced had a more significant gingival recession than those who had not. No discernible changes were found when comparing the two groups for clinical attachment loss and pocket depth. Thirteen people who had piercings for more than four years had a significantly higher incidence of tooth and periodontal problems, suggesting a correlation between piercing length and dental malformations.	Tongue piercings are more common than lip piercings, and so are dental abnormalities. Tongue and lip piercings both cause gingival recession. Dental abnormalities, gingival recession, attachment loss, and probing depth of teeth next to pierced locations are more common, and the longer the period, the more severe tongue and lip piercings are worn. Gingival recession rates are related to the morphology of ornaments.
Gill et al. (2012)	Retrospective 6794 (27.78%)/ 17665 (72.22%)	Oral piercing injuries collected from 2002 to 2008	24459 (NA)	Oral	They analyzed by injury type, anatomic site, and mechanism of injury according to age, gender, and race.	Lip and tongue wounds accounted for 46%, while tooth injuries accounted for 10% of all reported cases. The leading causes of damage were infections (42%), followed by soft tissue puncture wounds (29%). Overgrown mucosa around oral piercings is the leading cause of emergency department visits (39%). The need for hospitalization was very uncommon.	Teenagers and young adults account for most oral piercing injuries seen in American hospital emergency rooms. Data collected nationally suggests that emergency room dentists should be prepared to treat hard and soft tissue issues resulting from oral piercings.
Firoozmand et al. (2009)	Case-series (506 (55.58%)/421 (45.45%))	Teenage students with oral piercing	927 (16.14 ± 1.03)	Oral	Clinical examinations were performed on all subjects, and a questionnaire was sent to gather information on demographics, including gender, piercing site, mouth problems or modifications, and frequency of cleaning.	Only 3.6% of the children evaluated had oral piercings, with 69.70% attending public schools and 30.30% attending private ones. A slight majority of men (54.55%) outnumbered girls (45.45%). Most people (66.6%) had their tongues pierced. In 74.3% of the instances, piercing led to the difficulties and modifications expected from its usage.	Oral piercing was only seen in 3.6% of high school students in the group investigated, and it was linked to some minor health issues in those who had it done.
Campbell et al. (2002)	Case-series (31 (60%)/21 (30%))	Adults with tongue piercings	52 (22 ± 5)	Tongue	Patients were examined for gingival recession on the lingual aspect of the 12 anterior teeth and tooth chipping anywhere in the mouth.	No one had lingual recession or tooth chipping within the group of people whose tongues were pierced between 0 and 2 years ago. Half of the participants who wore lengthy barbells for 2+ years had lingual regression on their mandibular central incisors. After four years or more of having their tongues pierced, 47% of patients had chipped molars or premolars.	The front mandibular teeth and the posterior molars are more likely to have lingual recession after a tongue piercing. These side effects are more common in those who use a tongue barbell often. Recession and chipping seem to affect barbells of varying stem lengths. Efforts to end tongue piercing should focus on young adults, who comprise most of those who acquire them.
Pires et al. (2010)	Case (27 (45%)/33 (55.5%)) control (43 (35.8%)/77 (64.2%))	Individuals with tongue piercings	60 (18.9 ± 3.9)/120 (17.78 ± 3.8)	Tongue	Periodontal characteristics and tooth fractures were among the factors considered in the clinical review of patients’ oral health records.	It was shown that gingival recession was more common and severe in the case group than in the control group. Tongue piercings were associated with an increased risk of gingival recession in the anterior lingual mandibular area by a factor of 11 compared to those without piercings. In the front lingual mandibular area, piercing usage, older age, male gender, and bleeding on probing were all linked with gingival recession.	There was a significant correlation between tongue piercings and gingival recession in the anterior lingual mandibular area.
Kieser et al. (2005)	Case-series (3 (7%)/40 (93%))	Adult dental patients with tongue and lip piercings	43 (21 ± 5)	Tongue and lip	A questionnaire was given to patients after they had undergone an intraoral examination.	76.7% of participants had their tongues pierced, 34.9% had their lips pierced, and 11.6% had both. Only 4 of the piercings had been performed by a medical professional—problems arising after a piercing were noted by 34.9% of patients. Gingival recession was present in at least one labial piercing site in eighty percent of those with them and almost one-third of those with tongue piercings. The chances of developing lingual recession increased by 1.17 for every year beyond the age of 14; this trend was statistically significant. While age did predict the incidence of labial recession and the number of afflicted sites, it did not predict the number of lingual sites with recession. No correlations between piercings and either abnormal tooth wear or damage were found.	These results indicate a possible link between oral piercings and localized gingival recession, suggesting that patients should be warned of the potential risks to their periodontal health as part of the informed consent process.
Levin et al. (2005)	Case-series (210 (54%)/179 (46%))	Young adults with oral piercing	389 (20.08 ± 1.1)	Oral	Patients were given a questionnaire on oral piercing, complication awareness, and piercing-related complication incidence before their dental checkups.	Lingual piercing was the most prevalent form of oral piercing, with 79 individuals (20.3%) reporting they had one. Forty-one (51.9%) and thirty-six (45.7%) individuals experienced swelling and bleeding after piercing. Of the whole sample, 225 people (57.8%) had no idea that having an intra-oral piercing may be harmful. On closer inspection, 15 teeth were broken in 11 (or 13.9%) of the pierces. Twenty-one patients (or 26.6%) were found to have gingival recessions, most often around the mandibular incisors.	As the number of patients with pierced intra- and perioral locations rises, dentists should be prepared to advise their patients on the risks and benefits of this body modification.
De Moor et al. (2000)	Cross-sectional Study (NA)	Patients with tongue-piercing	15 (NA)	Body of the tongue, anterior to the lingual frenum	Individuals were evaluated based on clinical and radiological criteria	Teeth chipping was reported as the most prevalent dental issue. In addition, there were two broken teeth and four broken cusps. Selective dental abrasion was reported once—the majority of patients presented with trauma to the anterior lingual gingiva. Only 2 of the 15 polled people reported experiencing saliva flow. Patients did not report any difficulties with eating, chewing, or swallowing. A single incident of galvanic current generation due to the appliance was reported.	Based on the collected data, it was concluded that the dentistry community should play a more significant role in informing patients about tongue and oral piercing risks.
De Moor et al. (2005)	Case-series (13 (26%)/37 (74%))	Patients with oral piercing	50 (NA)	Oral and perioral	Individuals were evaluated based on clinical and radiological criteria	Chipped teeth were the most often reported dental issue, and they were most prevalent after getting a tongue piercing. Lip piercing with studs was associated with gingival regression. Oedema, bleeding, and infection were some of the postoperative problems.	More power should be provided to dentists and oral and maxillofacial surgeons to counsel patients who have or are considering oral and facial piercings.

**Table 2:** The quality assessment of case–control studies using the Newcastle–Ottawa Scale (NOS).

Studies	Selection	Comparability	Outcome	Score
Ibraheem et al. (2022)	★★★★	★	★★	7
Schmidt et al. (2019)	★★★★	★★	★★	8
Ziebolz et al. (2019)	★★★★	★	★★	7
Ziebolz et al. (2020)	★★★★	★★	★★	8
Ziebolz et al. (2012)	★★	★★	★★	6
Zadik et al. (2010)	★★	★	★★	5
Ventä et al. (2005)	★★	★★	★★	6
Giuca et al. (2012)	★★	★★	★★	6
Pires et al. (2010)	★★★	★★	★★	7

Note: If a study fulfilled the criteria for an item, a ★ was awarded. A maximum of ★★★★ was possible within the selection category; two stars were given for comparability, and a maximum of three stars for each item was possible in the exposure category. The total score, therefore, ranges from zero to nine, with higher scores indicating a lower risk of bias. When all items were well reported, there was a low risk of bias (6–9 stars). When items were not reported, unclear, or insufficient, there was a moderate (4–5 points) or high (1–3 points) risk of bias.

**Table 3:** The quality assessment of cohort studies using the Newcastle–Ottawa Scale (NOS).

Studies	Selection	Comparability	Outcome	Score
Inchingolo et al. (2011)	★★★	★	★★	6
Gill et al. (2012)	★★★★	★★	★★	8

Note: If a study fulfilled the criteria for an item, a ★ was awarded. A maximum of ★★★★ was possible within the selection category; two stars were given for comparability, and a maximum of three stars for each item was possible in the exposure category. The total score, therefore, ranges from zero to nine, with higher scores indicating a lower risk of bias. When all items were well reported, there was a low risk of bias (6–9 stars). When items were not reported, unclear, or insufficient, there was a moderate (4–5 points) or high (1–3 points) risk of bias.

**Table 4:** The quality assessment of cross-sectional studies using the Newcastle–Ottawa Scale (NOS).

Studies	Selection	Comparability	Outcome	Score
Vilchez-Perez et al. (2009)	★★	★	★★	5
Lorenzini et al. (2008)	★★★	★★	★★★	8
Kapferer et al. (2012)	★★★★	★★	★★	8
De Moor et al. (2000)	★★	★	★	4

Note: If a study fulfilled the criteria for an item, a ★ was awarded. A maximum of ★★★★ was possible within the selection category; two stars were given for comparability, and a maximum of three stars for each item was possible in the exposure category. The total score, therefore, ranges from zero to nine, with higher scores indicating a lower risk of bias. When all items were well reported, there was a low risk of bias (6–9 stars). When items were not reported, unclear, or insufficient, there was a moderate (4–5 points) or high (1–3 points) risk of bias.

**Table 5:** Quality assessment of case series studies using the Quality Appraisal Checklist of the Institute of Health Economics (IHE).

	Study Objective	Study Design			Study Population			Intervention and Co-Intervention		Outcome Measure				Statistical Analysis	Results and Conclusions						**Estimated Risk of Bias**
QN	1	2	3	4	5	6	7	8	9	10	11	12	13	14	15	**16**	**17**	**18**	**19**	**20**	
Study																					
Covello et al. (2020)	+	+	+	+	+	+	+	NA	NA	+	NA	+	NA	+	+	NA	+	+	+	+	low
King et al. (2018)	+	+	?	+	+	+	+	NA	NA	+	NA	+	NA	+	+	NA	+	+	+	+	low
Mejersjo et al. (2016)	+	+	+	+	+	+	+	NA	NA	+	NA	+	NA	+	+	NA	+	+	+	+	low
Schmidt et al. (2019)	+	+	+	+	+	+	+	NA	NA	+	NA	+	NA	+	+	NA	+	+	+	+	low
Vozza et al. (2014)	+	+	-	+	+	+	+	NA	NA	+	NA	+	NA	+	+	NA	+	+	+	+	low
Ziebolz et al. (2009)	+	+	-	+	+	+	+	NA	NA	+	NA	+	NA	+	+	NA	+	+	+	-	low
Vieira et al. (2011)	+	+	+	+	-	+	+	NA	NA	+	NA	+	NA	+	+	NA	+	+	+	-	low
Saquet et al. (2009)	+	+	+	+	?	+	+	NA	NA	+	NA	+	NA	+	+	NA	?	+	+	+	low
Pearose et al. (2006)	+	+	+	+	+	+	+	NA	NA	+	NA	+	NA	+	+	NA	+	+	+	+	low
Oberholzer et al. (2010)	+	+	+	+	+	-	+	NA	NA	+	NA	+	NA	+	+	NA	+	+	+	+	low
López-Jornet et al. (2006)	+	+	-	+	+	+	+	NA	NA	+	NA	+	NA	+	+	NA	+	+	+	+	low
López-Jornet et al. (2006) (2)	+	+	-	+	+	+	+	NA	NA	+	NA	+	NA	+	+	NA	+	+	+	-	low
Kapferer et al. (2011)	+	+	+	+	?	+	+	NA	NA	+	NA	+	NA	+	+	NA	+	+	+	-	low
Hickey et al. (2010)	+	+	+	+	+	+	+	NA	NA	+	NA	+	NA	+	+	NA	+	+	+	+	low
Firoozmand et al. (2009)	+	+	-	+	+	-	+	NA	NA	+	NA	+	NA	-	-	NA	-	-	+	?	high
Campbell et al. (2002)	+	+	+	-	-	+	+	NA	NA	+	NA	+	NA	-	+	NA	-	+	+	+	moderate
Kieser et al. (2005)	+	+	-	-	+	-	+	NA	NA	+	NA	?	NA	+	+	NA	-	+	+	-	moderate
Levin et al. (2005)	+	+	+	+	+	-	+	NA	NA	+	NA	+	NA	+	+	NA	-	+	?	-	moderate
De Moor et al. (2005)	+	+	+	+	-	+	+	NA	NA	?	NA	?	NA	-	+	NA	+	+	+	-	moderate

+, Yes (Green);-, No (Red); ?, Unclear (Yellow); NA, not applicable. The evaluation questions for the checklist are enumerated as follows: 1. Was the study’s hypothesis/aim/objective clearly stated? 2. Was the study conducted prospectively? 3. Were the cases collected in more than one center? 4. Were patients recruited consecutively? 5. Were the characteristics of the patients included in the study described? 6. Were the eligibility criteria (i.e., inclusion and exclusion criteria) for entry into the study clearly stated? 7. Did patients enter the study at a similar point in the disease? 8. Was the intervention of interest clearly described? 9. Were additional interventions (co-interventions) clearly described? 10. Were relevant outcome measures established a priori? 11. Were outcome assessors blinded to the intervention that patients received? 12. Were the relevant outcomes measured using appropriate objective/subjective methods? 13. Were the relevant outcome measures made before and after the intervention? 14. Were the statistical tests used to assess the relevant outcomes appropriate? 15. Was follow-up long enough for important events to occur? 16. Were losses to follow-up reported? 17. Did the study provide estimates of random variability in the data analysis of the relevant outcomes? 18. Were the adverse events reported? 19. Were the study’s conclusions supported by results? 20. Were both competing interests and sources of support for the study reported?

## Data Availability

The data used in this study are available on request from the corresponding author.

## Supplementary Materials

The following supporting information can be downloaded at https://www.mdpi.com/article/10.3390/diagnostics13213371/s1, Table S1: An example of the search strategy used for the databases search.
