# Supplementary material for: Oral Complications Associated with the Piercing of Oral and Perioral Tissues and the Corresponding Degree of Awareness among Public and Professionals: A Systematic Review
Source: Diagnostics (Basel). 2023 Nov 2;13(21):3371. doi: 10.3390/diagnostics13213371 (PMC10647284; doi:10.3390/diagnostics13213371)
Supplement: Supplementary file 1 [file diagnostics-13-03371-s001.zip › diagnostics-2688955-supplementary.pdf]

Supplementary Table S1. An example of the search strategy used for the databases search.

**Search Strategy**	{[<piercing* OR pierce*> AND <mouth OR oral OR lip* OR labret* OR cheek* OR bucca* OR tongue OR lingua* OR frenulum* OR philtrum* OR uvul* OR venom OR tooth OR gingiva* OR gum* OR mucos*>] OR [(mouth piercing*) OR (oral piercing) OR (lip piercing) OR (cheek piercing) OR (buccal piercing*) OR (tongue piercing) OR (lingual piercing*) OR (frenulum piercing) OR (uvula piercing) OR (venom piercing) OR (tooth piercing*) OR (gingiva piercing) OR (gums piercing) OR (mucosa piercing)]}.
